# 1-[4-Chloro-3-(trifluoro­meth­yl)phen­yl]-4-phenyl-1*H*-1,2,3-triazole

**DOI:** 10.1107/S1600536812042705

**Published:** 2012-10-20

**Authors:** Jarrad M. Altimari, Peter C. Healy, Luke C. Henderson

**Affiliations:** a SRC for Biotechnology, Chemistry and Systems Biology, Faculty of Science and Technology, Deakin University, Vic, 3216, Australia; b Queensland Micro and Nanotechnology Centre, Griffith University, Brisbane 4111, Australia; c Institute for Frontier Materials, SRC for Biotechnology, Chemistry and Systems Biology, Faculty of Science and Technology, Deakin University, Vic, 3216, Australia

## Abstract

In the title compound, C_15_H_9_ClF_3_N_3_, the phenyl and chloro-trifluoro­methyl benzene rings are twisted with respect to the planar triazole group, making dihedral angles of 21.29 (12) and 32.19 (11)°, respectively. In the crystal, the mol­ecules pack in a head-to-tail arrangement along the *a* axis with closest inter-centroid distances between the triazole rings of 3.7372 (12) Å.

## Related literature

For background to the synthesis of *N*-aryl-1,2,3-triazoles, see: Bock *et al.* (2006 ▶); Irie *et al.* (2012 ▶). For biological background, see: Jia & Zhu (2010 ▶); Henderson *et al.* (2012 ▶); Alam *et al.* (2006 ▶, 2007 ▶). For related structures, see: Lin *et al.* (2008 ▶); Lin (2010 ▶).
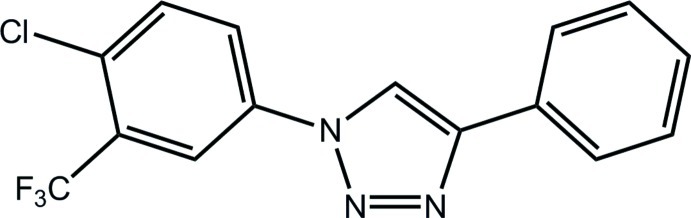


## Experimental

### 

#### Crystal data

C_15_H_9_ClF_3_N_3_
*M* *_r_* = 323.70
Monoclinic, 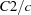

*a* = 30.7475 (16) Å
*b* = 5.8877 (3) Å
*c* = 15.4364 (8) Å
β = 105.470 (5)°
*V* = 2693.2 (2) Å^3^
*Z* = 8
Mo *K*α radiation
μ = 0.32 mm^−1^
*T* = 249 K
0.33 × 0.26 × 0.24 mm

#### Data collection

Oxford Diffraction GEMINI S Ultra diffractometer
Absorption correction: multi-scan (*CrysAlis PRO*; Agilent, 2012 ▶), *T*_min_ = 0.902, *T*_max_ = 0.928
3934 measured reflections
2355 independent reflections
1899 reflections with *I* > 2σ(*I*)
*R*_int_ = 0.021

#### Refinement

*R*[*F* ^2^ > 2σ(*F* ^2^)] = 0.041
*wR*(*F* ^2^) = 0.091
*S* = 1.07
2355 reflections
199 parameters
H-atom parameters constrained
Δρ_max_ = 0.18 e Å^−3^
Δρ_min_ = −0.22 e Å^−3^

### 

Data collection: *CrysAlis PRO* (Agilent, 2012 ▶); cell refinement: *CrysAlis PRO* (Agilent, 2012 ▶); data reduction: *CrysAlis PRO*; program(s) used to solve structure: *TEXSAN* (Molecular Structure Corporation, 2001 ▶) and *SIR97* (Altomare *et al.*, 1999 ▶); program(s) used to refine structure: *TEXSAN* and *SHELXL97* (Sheldrick, 2008 ▶); molecular graphics: *ORTEP-3 for Windows* (Farrugia, 1997 ▶); software used to prepare material for publication: *PLATON* (Spek, 2009 ▶).

## Supplementary Material

Crystal structure: contains datablock(s) global, I. DOI: 10.1107/S1600536812042705/tk5159sup1.cif

Structure factors: contains datablock(s) I. DOI: 10.1107/S1600536812042705/tk5159Isup2.hkl

Supplementary material file. DOI: 10.1107/S1600536812042705/tk5159Isup3.cml

Additional supplementary materials:  crystallographic information; 3D view; checkCIF report

## Figures and Tables

**Fig. 1.**
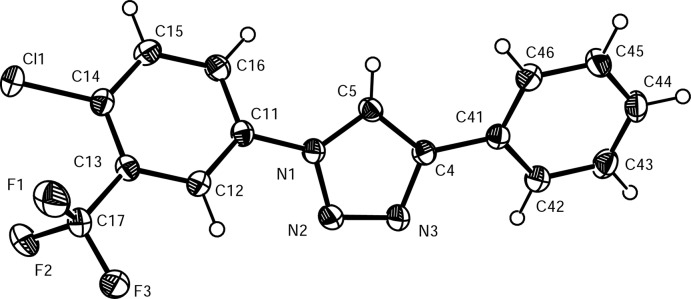
The molecular structure of the title compound with atom labelling and displacement ellipsoids for non-H atoms drawn at the 40% probability level. Hydrogen atoms are shown as spheres of arbitrary radius.

**Fig. 2.**
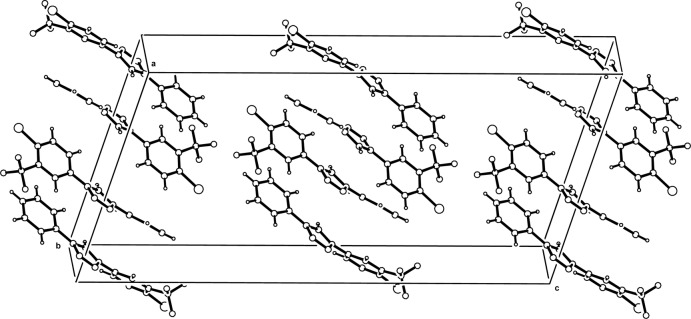
Molecular packing of the title compound viewed along [0 1 0].

## supplementary crystallographic information

### Comment

The structure of the title compound, (I), was determined as part of an ongoing project developing *N*-aryl-1,2,3-triazoles as amide mimetics for medicinal applications. The synthesis of 1,2,3-triazoles *via* copper mediated 1,3-dipolar reactions has become one of the most widely used methodologies to tether molecules together or to a surface (Bock *et al.*, 2006; Irie *et al.*, 2012). Electronically deactivated *N*-phenyl-1,2,3-triazoles have been employed in several areas such as the combinatorial development of kinase inhibitors (Jia & Zhu, 2010) in the development of monoamine oxidase inhibitors, androgen receptor antagonists (Henderson *et al.*, 2012) and as GABA receptor antagonists (Alam *et al.*, 2006; Alam *et al.*, 2007). This compound provides an aryl chloride moiety in the *para*-position relative to the triazole ring providing a synthetic handle for further structural elaboration.

In the molecular structure of (I) (Fig. 1) the planar phenyl and chloro-trifluoromethyl benzene rings are twisted with respect to the central planar triazole group with dihedral angles of 21.29 (12) and 32.19 (11)°, respectively. In the triazole ring, the N1—N2 and N2—N3 bond lengths are 1.357 (3) and 1.310 (3) Å, respectively. and are similar to those reported for related compounds (Lin *et al.*, 2008; Lin, 2010). In the crystal lattice, the molecules stack in a head to tail arrangement along the *a* axis (Fig. 2) with the centroid-centroid distances between the triazole rings 4.1494 (12) and 3.7372 (12) Å.

### Experimental

Phenyl acetylene (127 mg, 1.25 mmol, 1 eq), 4-azido-1-chloro-2-(trifluoromethyl) benzene (230 mg, 1.04 mmol, 1 eq) and copper(I) chloride (10 mg, 10 mol%) were stirred in water (3 ml) for 10 min. The solution was then stirred under microwave irradiation at 100°C for 30 min in a sealed vessel. The solution cooled to room temperature, dichloromethane (3 ml) was added and the biphasic mixture stirred for 3 min. The aqueous phase was then extracted using dichloromethane (2 *x* 10 ml), the combined organic layers were washed with HCl (4*M*, 5 ml), NaOH (1*M*, 5 ml), water (5 ml). The organic phase dried over MgSO_4_, filtered and concentrated under vacuum. The solution was then taken up in chloroform and allowed to slowly evaporate. ν (max) cm^-1^ 3124, 1495, 1310, 1147, 1037. ^1^H NMR (400 MHz, CDCl_3_): δ = 9.50 (1H, s, triazole H), 8.42 (1H, s, ArH), 8.32 (1H, dd, J = 8.7, 2.6 Hz, ArH), 8.02 (1H, d, J = 8.7 Hz, ArH), 7.95 (2H, d, J = 8 Hz, ArH), 7.51 (2H, m, ArH), 7.40 (1H, m, ArH). ^13^C NMR (100 MHz, CDCl_3_): δ = 148.22, 136.16, 133.98, 130.91 (*m*), 130.47, 129.65, 129.04, 128.53 (q, J^2^_C—F_ = 32 Hz), 125.93, 125.70 (*m*), 122.89 (q, J^1^_C—F_ = 272 Hz), 120.62, 119.75. *M*.pt: 443–445.3 K. HRMS, m/*z* calcd for(C_15_H_9_ClF_3_N_3_) 324.05099, found 324.05011.

### Refinement

The carbon-bound H atoms were constrained as riding with C—H = 0.95 Å, and with *U*_iso_(H) = 1.2*U*_eq_ of the parent C atom.

### Crystal data

**Table:**

C_15_H_9_ClF_3_N_3_	*F*(000) = 1312
*M**_r_* = 323.70	*D*_x_ = 1.597 Mg m^−^^3^
Monoclinic, *C*2/*c*	Mo *K*α radiation, λ = 0.71070 Å
Hall symbol: -C 2yc	Cell parameters from 1854 reflections
*a* = 30.7475 (16) Å	θ = 3.4–30.3°
*b* = 5.8877 (3) Å	µ = 0.32 mm^−^^1^
*c* = 15.4364 (8) Å	*T* = 249 K
β = 105.470 (5)°	Block, colourless
*V* = 2693.2 (2) Å^3^	0.33 × 0.26 × 0.24 mm
*Z* = 8	

### Data collection

**Table:**

Oxford Diffraction GEMINI S Ultra diffractometer	2355 independent reflections
Radiation source: Enhance (Mo) X-ray Source	1899 reflections with *I* > 2σ(*I*)
Graphite monochromator	*R*_int_ = 0.021
Detector resolution: 16.0774 pixels mm^-1^	θ_max_ = 25.0°, θ_min_ = 3.4°
ω and φ scans	*h* = −36→33
Absorption correction: multi-scan (*CrysAlis PRO*; Agilent, 2012),	*k* = −5→6
*T*_min_ = 0.902, *T*_max_ = 0.928	*l* = −9→18
3934 measured reflections	

### Refinement

**Table:**

Refinement on *F*^2^	Primary atom site location: structure-invariant direct methods
Least-squares matrix: full	Secondary atom site location: difference Fourier map
*R*[*F*^2^ > 2σ(*F*^2^)] = 0.041	Hydrogen site location: inferred from neighbouring sites
*wR*(*F*^2^) = 0.091	H-atom parameters constrained
*S* = 1.07	*w* = 1/[σ^2^(*F*_o_^2^) + (0.0327*P*)^2^ + 2.2063*P*] where *P* = (*F*_o_^2^ + 2*F*_c_^2^)/3
2355 reflections	(Δ/σ)_max_ = 0.001
199 parameters	Δρ_max_ = 0.18 e Å^−^^3^
0 restraints	Δρ_min_ = −0.22 e Å^−^^3^

### Special details

**Table:**

Geometry. Bond distances, angles *etc*. have been calculated using the rounded fractional coordinates. All su's are estimated from the variances of the (full) variance-covariance matrix. The cell e.s.d.'s are taken into account in the estimation of distances, angles and torsion angles
Refinement. Refinement of *F*^2^ against ALL reflections. The weighted *R*-factor *wR* and goodness of fit *S* are based on *F*^2^, conventional *R*-factors *R* are based on *F*, with *F* set to zero for negative *F*^2^. The threshold expression of *F*^2^ > σ(*F*^2^) is used only for calculating *R*-factors(gt) *etc*. and is not relevant to the choice of reflections for refinement. *R*-factors based on *F*^2^ are statistically about twice as large as those based on *F*, and *R*- factors based on ALL data will be even larger.

### Fractional atomic coordinates and isotropic or equivalent isotropic displacement parameters (Å^2^)

**Table:**

	*x*	*y*	*z*	*U*_iso_*/*U*_eq_	
Cl1	0.78052 (2)	0.33228 (12)	0.32573 (4)	0.0454 (2)	
F1	0.76886 (4)	0.0366 (3)	0.48769 (11)	0.0575 (6)	
F2	0.78035 (5)	−0.1587 (3)	0.37911 (10)	0.0587 (5)	
F3	0.81441 (4)	−0.2452 (3)	0.51428 (10)	0.0522 (5)	
N1	0.96027 (6)	0.1414 (3)	0.56964 (11)	0.0285 (6)	
N2	0.97402 (6)	−0.0774 (3)	0.58511 (13)	0.0369 (6)	
N3	1.01508 (6)	−0.0729 (3)	0.63832 (12)	0.0358 (6)	
C4	1.02780 (7)	0.1481 (4)	0.65769 (13)	0.0276 (7)	
C5	0.99302 (7)	0.2849 (4)	0.61383 (14)	0.0281 (7)	
C11	0.91648 (7)	0.1919 (4)	0.51321 (13)	0.0275 (6)	
C12	0.88165 (7)	0.0422 (4)	0.51110 (13)	0.0281 (7)	
C13	0.83879 (7)	0.0861 (4)	0.45597 (13)	0.0277 (7)	
C14	0.83212 (7)	0.2782 (4)	0.40213 (13)	0.0293 (7)	
C15	0.86683 (7)	0.4302 (4)	0.40630 (14)	0.0335 (7)	
C16	0.90929 (7)	0.3881 (4)	0.46234 (14)	0.0324 (7)	
C17	0.80065 (7)	−0.0695 (4)	0.45873 (15)	0.0365 (8)	
C41	1.07225 (7)	0.2101 (4)	0.71678 (13)	0.0276 (7)	
C42	1.10818 (7)	0.0584 (4)	0.73080 (14)	0.0344 (7)	
C43	1.15027 (7)	0.1168 (5)	0.78475 (15)	0.0394 (8)	
C44	1.15723 (8)	0.3258 (5)	0.82582 (15)	0.0398 (8)	
C45	1.12182 (8)	0.4780 (5)	0.81243 (14)	0.0389 (8)	
C46	1.07966 (7)	0.4216 (4)	0.75828 (14)	0.0333 (7)	
H5	0.99210	0.43430	0.61430	0.0340*	
H12	0.88700	−0.09090	0.54740	0.0330*	
H15	0.86140	0.56400	0.37040	0.0400*	
H16	0.93320	0.49300	0.46580	0.0390*	
H42	1.10380	−0.08670	0.70260	0.0410*	
H43	1.17450	0.01160	0.79350	0.0470*	
H44	1.18610	0.36500	0.86340	0.0480*	
H45	1.12650	0.62270	0.84080	0.0470*	
H46	1.05570	0.52790	0.74940	0.0400*	

### Atomic displacement parameters (Å^2^)

**Table:**

	*U*^11^	*U*^22^	*U*^33^	*U*^12^	*U*^13^	*U*^23^
Cl1	0.0334 (3)	0.0438 (4)	0.0481 (3)	0.0047 (3)	−0.0079 (3)	0.0048 (3)
F1	0.0322 (7)	0.0558 (11)	0.0906 (11)	−0.0039 (8)	0.0271 (8)	−0.0025 (9)
F2	0.0579 (9)	0.0458 (10)	0.0574 (9)	−0.0175 (8)	−0.0109 (7)	−0.0089 (8)
F3	0.0371 (8)	0.0447 (10)	0.0679 (9)	−0.0101 (7)	0.0019 (7)	0.0210 (8)
N1	0.0223 (9)	0.0286 (11)	0.0336 (9)	0.0001 (8)	0.0055 (7)	0.0002 (8)
N2	0.0283 (10)	0.0288 (12)	0.0489 (11)	−0.0018 (9)	0.0020 (9)	−0.0005 (9)
N3	0.0257 (9)	0.0317 (12)	0.0447 (11)	0.0000 (9)	0.0003 (8)	0.0003 (9)
C4	0.0243 (11)	0.0294 (13)	0.0300 (11)	−0.0012 (10)	0.0087 (9)	0.0004 (10)
C5	0.0246 (11)	0.0266 (13)	0.0331 (11)	−0.0036 (10)	0.0077 (9)	−0.0017 (10)
C11	0.0225 (10)	0.0309 (13)	0.0288 (10)	−0.0002 (10)	0.0066 (9)	−0.0014 (10)
C12	0.0260 (11)	0.0294 (13)	0.0280 (10)	0.0024 (10)	0.0058 (9)	0.0011 (10)
C13	0.0255 (11)	0.0288 (13)	0.0286 (11)	−0.0020 (10)	0.0068 (9)	−0.0048 (9)
C14	0.0264 (11)	0.0326 (14)	0.0276 (11)	0.0034 (10)	0.0051 (9)	−0.0022 (10)
C15	0.0341 (12)	0.0321 (14)	0.0346 (12)	0.0041 (11)	0.0096 (10)	0.0064 (10)
C16	0.0274 (11)	0.0332 (14)	0.0372 (12)	−0.0039 (10)	0.0099 (10)	0.0015 (10)
C17	0.0263 (11)	0.0368 (15)	0.0419 (13)	−0.0019 (11)	0.0012 (10)	0.0003 (12)
C41	0.0262 (11)	0.0310 (13)	0.0259 (10)	−0.0011 (10)	0.0075 (9)	0.0030 (10)
C42	0.0306 (11)	0.0335 (14)	0.0373 (12)	0.0007 (11)	0.0059 (10)	0.0005 (11)
C43	0.0284 (12)	0.0453 (17)	0.0417 (13)	0.0045 (12)	0.0044 (10)	0.0083 (12)
C44	0.0305 (12)	0.0508 (17)	0.0324 (12)	−0.0082 (13)	−0.0016 (10)	0.0047 (12)
C45	0.0422 (13)	0.0381 (15)	0.0323 (12)	−0.0082 (12)	0.0026 (10)	−0.0042 (11)
C46	0.0332 (12)	0.0345 (14)	0.0315 (11)	0.0031 (11)	0.0076 (10)	0.0002 (10)

### Geometric parameters (Å, º)

**Table:**

Cl1—C14	1.735 (2)	C15—C16	1.383 (3)
F1—C17	1.334 (3)	C41—C42	1.392 (3)
F2—C17	1.329 (3)	C41—C46	1.391 (3)
F3—C17	1.338 (3)	C42—C43	1.383 (3)
N1—N2	1.357 (3)	C43—C44	1.375 (4)
N1—C5	1.352 (3)	C44—C45	1.382 (4)
N1—C11	1.426 (3)	C45—C46	1.383 (3)
N2—N3	1.310 (3)	C5—H5	0.8800
N3—C4	1.369 (3)	C12—H12	0.9500
C4—C5	1.366 (3)	C15—H15	0.9500
C4—C41	1.473 (3)	C16—H16	0.9500
C11—C12	1.381 (3)	C42—H42	0.9500
C11—C16	1.381 (3)	C43—H43	0.9500
C12—C13	1.389 (3)	C44—H44	0.9500
C13—C14	1.386 (3)	C45—H45	0.9500
C13—C17	1.498 (3)	C46—H46	0.9500
C14—C15	1.381 (3)		
			
Cl1···F1	3.1445 (18)	C41···H15^x^	3.0300
Cl1···F2	3.0063 (19)	C42···H45^vi^	3.0400
Cl1···F2^i^	3.1086 (19)	C42···H15^x^	3.0100
Cl1···F2^ii^	3.2167 (16)	C43···H15^x^	2.9900
F1···Cl1	3.1445 (18)	C44···H15^x^	3.0000
F1···F1^iii^	2.835 (2)	C45···H42^i^	3.0400
F1···F3^iv^	3.075 (2)	C45···H15^x^	3.0100
F2···Cl1^v^	3.2167 (16)	C46···H5	3.0000
F2···Cl1^vi^	3.1085 (19)	C46···H15^x^	3.0300
F2···Cl1	3.0063 (19)	H5···N2^i^	2.9400
F3···C45^vii^	3.295 (3)	H5···C16	2.9800
F3···C15^vi^	3.235 (3)	H5···C46	3.0000
F3···F1^iv^	3.075 (2)	H5···H16	2.5400
F1···H44^viii^	2.8100	H5···H46	2.5100
F3···H45^vii^	2.6000	H12···F3	2.3400
F3···H12	2.3400	H12···N2	2.5800
N2···H5^vi^	2.9400	H12···H45^vii^	2.5300
N2···H12	2.5800	H15···C41^x^	3.0300
N3···H42	2.6400	H15···C42^x^	3.0100
C5···C46^ix^	3.449 (3)	H15···C43^x^	2.9900
C12···C43^ix^	3.569 (3)	H15···C44^x^	3.0000
C12···C44^ix^	3.487 (3)	H15···C45^x^	3.0100
C15···C45^x^	3.527 (3)	H15···C46^x^	3.0300
C15···C46^x^	3.486 (3)	H16···C5	2.8000
C15···F3^i^	3.235 (3)	H16···H5	2.5400
C43···C12^ix^	3.569 (3)	H42···N3	2.6400
C44···C12^ix^	3.487 (3)	H42···C45^vi^	3.0400
C45···F3^xi^	3.295 (3)	H42···C14^xii^	3.0800
C45···C15^x^	3.527 (3)	H42···C15^xii^	2.9200
C46···C5^ix^	3.449 (3)	H42···C16^xii^	3.0400
C46···C15^x^	3.486 (3)	H44···F1^xiii^	2.8100
C5···H16	2.8000	H45···C42^i^	3.0400
C5···H46	2.8300	H45···F3^xi^	2.6000
C14···H42^xii^	3.0800	H45···H12^xi^	2.5300
C15···H42^xii^	2.9200	H46···C5	2.8300
C16···H42^xii^	3.0400	H46···H5	2.5100
C16···H5	2.9800		
			
N2—N1—C5	110.45 (18)	C4—C41—C42	120.3 (2)
N2—N1—C11	120.29 (18)	C4—C41—C46	121.2 (2)
C5—N1—C11	129.26 (19)	C42—C41—C46	118.5 (2)
N1—N2—N3	107.10 (17)	C41—C42—C43	120.7 (2)
N2—N3—C4	109.13 (17)	C42—C43—C44	120.4 (2)
N3—C4—C5	108.19 (19)	C43—C44—C45	119.5 (2)
N3—C4—C41	122.3 (2)	C44—C45—C46	120.6 (2)
C5—C4—C41	129.5 (2)	C41—C46—C45	120.4 (2)
N1—C5—C4	105.1 (2)	N1—C5—H5	127.00
N1—C11—C12	118.77 (19)	C4—C5—H5	127.00
N1—C11—C16	120.2 (2)	C11—C12—H12	120.00
C12—C11—C16	121.0 (2)	C13—C12—H12	120.00
C11—C12—C13	119.9 (2)	C14—C15—H15	120.00
C12—C13—C14	118.9 (2)	C16—C15—H15	120.00
C12—C13—C17	119.4 (2)	C11—C16—H16	120.00
C14—C13—C17	121.63 (19)	C15—C16—H16	120.00
Cl1—C14—C13	121.30 (17)	C41—C42—H42	120.00
Cl1—C14—C15	117.89 (17)	C43—C42—H42	120.00
C13—C14—C15	120.8 (2)	C42—C43—H43	120.00
C14—C15—C16	120.1 (2)	C44—C43—H43	120.00
C11—C16—C15	119.1 (2)	C43—C44—H44	120.00
F1—C17—F2	106.84 (18)	C45—C44—H44	120.00
F1—C17—F3	106.38 (18)	C44—C45—H45	120.00
F1—C17—C13	111.87 (19)	C46—C45—H45	120.00
F2—C17—F3	106.10 (19)	C41—C46—H46	120.00
F2—C17—C13	113.17 (18)	C45—C46—H46	120.00
F3—C17—C13	112.02 (18)		
			
C5—N1—N2—N3	−0.2 (2)	C11—C12—C13—C17	−176.1 (2)
C11—N1—N2—N3	179.82 (18)	C12—C13—C14—C15	−3.3 (3)
N2—N1—C5—C4	−0.1 (2)	C17—C13—C14—Cl1	−7.2 (3)
C11—N1—C5—C4	179.94 (19)	C12—C13—C17—F1	116.4 (2)
N2—N1—C11—C12	32.1 (3)	C12—C13—C17—F2	−122.9 (2)
N2—N1—C11—C16	−148.4 (2)	C17—C13—C14—C15	174.3 (2)
C5—N1—C11—C12	−147.9 (2)	C12—C13—C14—Cl1	175.22 (16)
C5—N1—C11—C16	31.6 (3)	C14—C13—C17—F2	59.6 (3)
N1—N2—N3—C4	0.4 (2)	C14—C13—C17—F3	179.48 (19)
N2—N3—C4—C5	−0.4 (2)	C12—C13—C17—F3	−3.0 (3)
N2—N3—C4—C41	179.51 (19)	C14—C13—C17—F1	−61.2 (3)
C41—C4—C5—N1	−179.6 (2)	Cl1—C14—C15—C16	−176.31 (17)
N3—C4—C5—N1	0.3 (2)	C13—C14—C15—C16	2.2 (3)
C5—C4—C41—C42	−158.3 (2)	C14—C15—C16—C11	0.5 (3)
C5—C4—C41—C46	20.4 (3)	C4—C41—C42—C43	178.7 (2)
N3—C4—C41—C46	−159.5 (2)	C42—C41—C46—C45	−0.3 (3)
N3—C4—C41—C42	21.9 (3)	C46—C41—C42—C43	0.0 (3)
C16—C11—C12—C13	1.2 (3)	C4—C41—C46—C45	−179.0 (2)
N1—C11—C12—C13	−179.29 (19)	C41—C42—C43—C44	0.3 (3)
N1—C11—C16—C15	178.24 (19)	C42—C43—C44—C45	−0.3 (4)
C12—C11—C16—C15	−2.2 (3)	C43—C44—C45—C46	0.0 (4)
C11—C12—C13—C14	1.6 (3)	C44—C45—C46—C41	0.3 (3)

Symmetry codes: (i) *x*, *y*+1, *z*; (ii) −*x*+3/2, *y*+1/2, −*z*+1/2; (iii) −*x*+3/2, −*y*+1/2, −*z*+1; (iv) −*x*+3/2, −*y*−1/2, −*z*+1; (v) −*x*+3/2, *y*−1/2, −*z*+1/2; (vi) *x*, *y*−1, *z*; (vii) −*x*+2, *y*−1, −*z*+3/2; (viii) *x*−1/2, −*y*+1/2, *z*−1/2; (ix) −*x*+2, *y*, −*z*+3/2; (x) −*x*+2, −*y*+1, −*z*+1; (xi) −*x*+2, *y*+1, −*z*+3/2; (xii) −*x*+2, −*y*, −*z*+1; (xiii) *x*+1/2, −*y*+1/2, *z*+1/2.
